# Do not stumble over the same “stone” twice: a case series of endogenous endophthalmitis secondary to severe systemic diseases

**DOI:** 10.1186/s12886-024-03478-7

**Published:** 2024-05-17

**Authors:** Ying He, Weijuan Zeng, Wenjian Shi, Xiaomin Chen, Yanru Shen, Shun Wang, Xiaojun Cai, Yang Liu, Yingying Gao, Min Ke

**Affiliations:** 1 Department of Ophthalmology, Zhongnan Hospital of Wuhan University, Wuhan, Hubei 430071 China; 2 Department of Ophthalmology, the Second Affiliated Hospital of Fujian Medical University, Quanzhou, Fujian 362000 China

**Keywords:** Endogenous endophthalmitis, C. Albicans, K. pneumoniae, Holmium:YAG laser lithotripsy, Pregnancy, Invasive liver abscess syndrome

## Abstract

**Background:**

Endogenous endophthalmitis (EE) is a rare but highly destructive eye emergency secondary to systemic infection. Acute endophthalmitis can lead to irreversible vision impairment or even loss of the whole eye, unless being diagnosed and treated promptly.

**Case presentation:**

This study reports three typical EE cases of endogenous endophthalmitis secondary to different severe systemic diseases. Patients were recruited from the Department of ophthalmology at Zhongnan hospital of Wuhan University and the Department of ophthalmology at the Second Affiliated Hospital of Fujian Medical University. Patients were followed up for up to 60 days. Among these cases, the eye symptoms is the initial manifestations while secondary to original different special systemic conditions. Patients have been treated under dynamically prompt response undergoing systemic treatment and eye treatment at the same time. Best corrected visual acuity were 20/40, 20/60 and light perception during follow-up evaluation.

**Conclusions:**

Our observation suggest that prompt identification and treatment could save patients’ vision from EE.

**Supplementary Information:**

The online version contains supplementary material available at 10.1186/s12886-024-03478-7.

## Background

Invasive procedures, including surgery, are extremely important to healthcare. It is reported that there are at least 230 million procedures performed annually worldwide and a steady increase in the number of procedures was observed over time [1, 2]. Unfortunately, whenever an invasive procedure is taken, there is a potential for micro-organisms to enter and spread through the body. Hematogenous spread of micro-organisms could cause a vision-threatening ophthalmic emergency called endogenous endophthalmitis (EE) [3]. Because of increased number of invasive procedures during clinical practice, the cases of EE are gradually increasing [4]. A recent study of the Nationwide Emergency Department (NEDS) Database indicated that the incidence of EE increased significantly from 0.10 per 100,000 in 2006 to 0.25 in 2017 in the US population [4]. 

EE accounts for approximately 2–8% of all endophthalmitis, which is characterized by prominent inflammation of the whole intraocular tissue [5, 6]. The main symptoms of endophthalmitis include decreased or lost vision, eye pain, eye redness, eyelids swollen, photophobia and floaters [5, 7]. Because eye tissues are very delicate, acute endophthalmitis can lead to severe irreversible vision impairment or even loss of the whole eye, unless being diagnosed and treated promptly. Early recognition of the disease and multidisciplinary collaboration could significantly impact the visual outcomes and quality of life of the patients [3]. Because EE is usually caused by systemic pathologies, the patients are often in a general non-ophthalmological condition. Non-ophthalmology colleagues are actually playing a crucial role in educating the patients of the possibility of the disease and watching for the early signs of potential EE, while ophthalmologists could only influence the choice of the proper therapy [8]. However, it is often a diagnostic challenge because colleagues in other departments may not be experienced in identifying EE, especially when those eye symptoms are still latent.

Here we present 3 cases, and all these patients had EE accompanying different systemic conditions. Our goal is to draw attention of our non-ophthalmology colleagues to this devastating eye disease.

## Case series

### Case 1

**Fig. 1:**
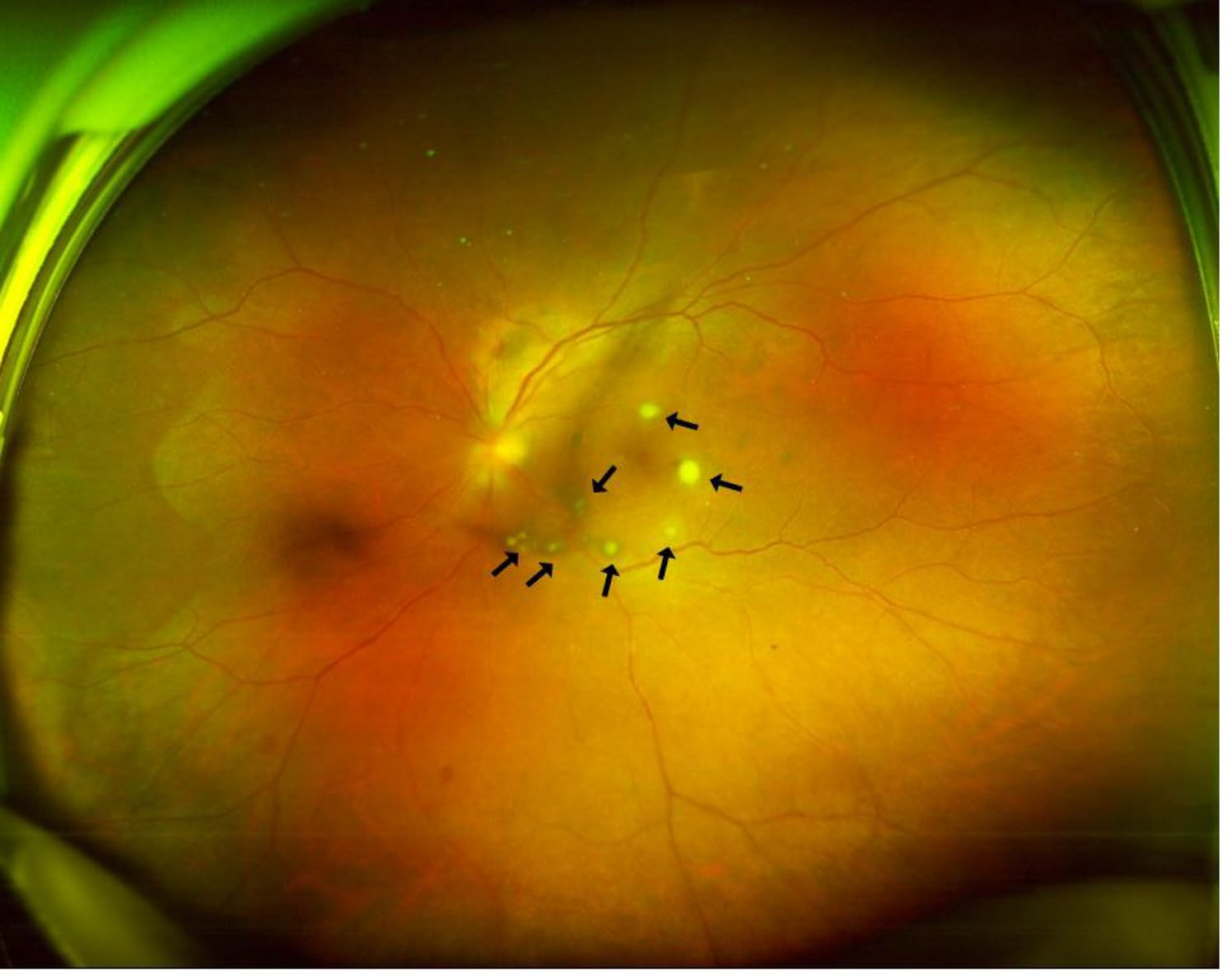
Fundus photograph of the left eye of the patient in case 1. Arrows indicate yellowish-white, circumscribed lesions on the surface of retina

**Fig. 2:**
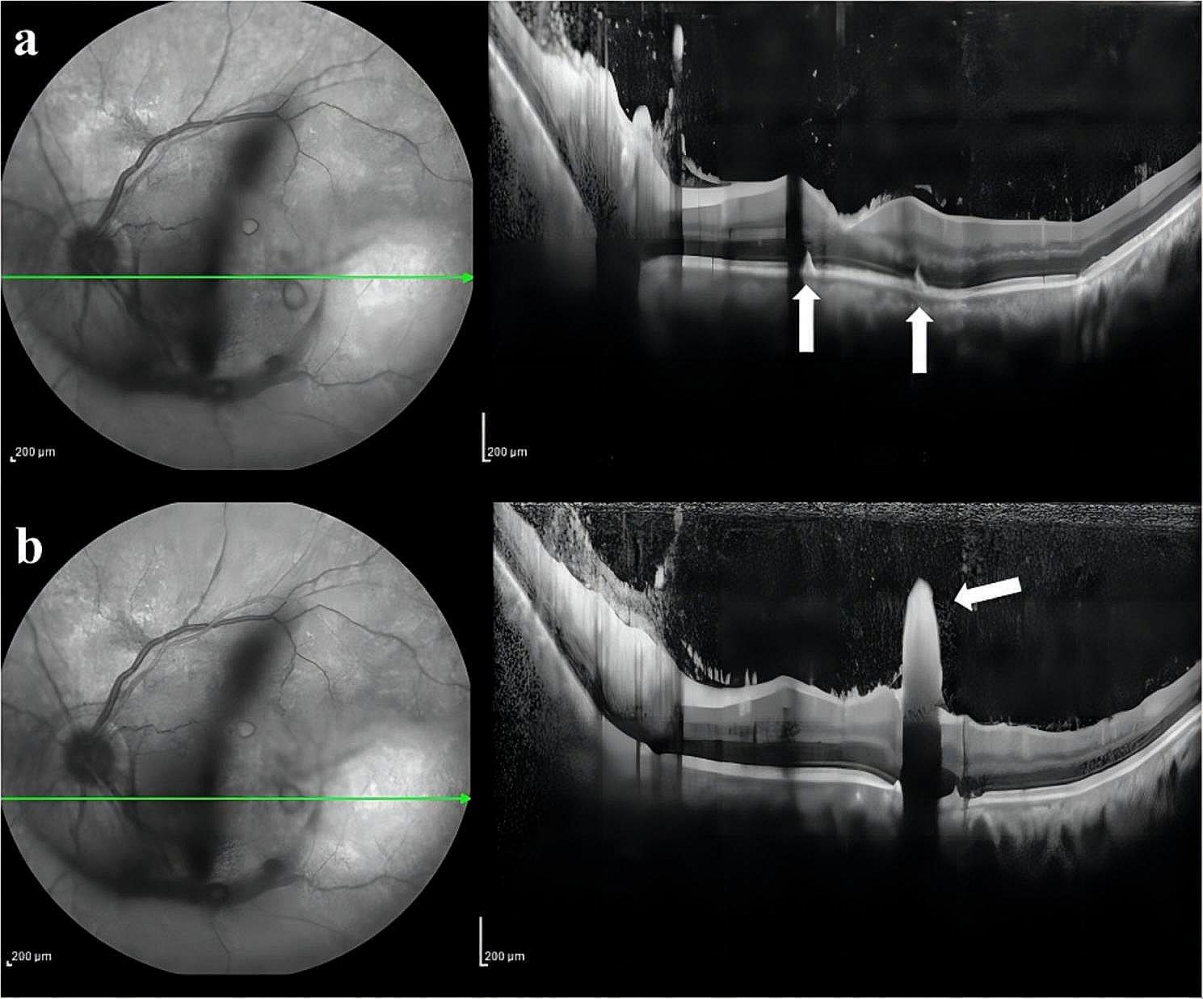
OCT scan of the circumscribed lesions on the retina. (**a**) Arrows indicate the lesions infiltrated from choroid into the retinal layers. (**b**) The arrow shows the lesion protruding from retinal layers into the vitreous

A 46-year-old male patient was referred to the ophthalmology department with blurred vision and floaters in his left eye for 7 days. His best corrected visual acuity was 20/20 (right) and 20/200 (left). The left eye showed marked conjunctival redness and vitreous inflammation, and intravitreal “puff ball” abscesses were observed. Fundus photograph showing dispersed multiple small, yellowish-white, circumscribed chorioretinal lesions (Fig. 1, Daytona P200T, Optos, Dunfermline, UK). The optical coherence tomography (OCT) findings indicated that chorioretinal lesion infiltrated from choroid into the retinal layers, and protruding into the vitreous (Fig. 2, Cirrus HD-OCT, Carl Zeiss Meditec, Dublin, CA). Intravitreal Examination of the fellow eye was unremarkable. The patient was diagnosed with urinary tract infection and left ureteral stone, and underwent uneventful ureteroscopy and holmium: YAG laser lithotripsy (HLL) in the same hospital 2 weeks before presentation to the ophthalmology department. His past medical and drug history was negative.

Diagnostic vitreous aspiration needle tap and anterior chamber paracentesis were performed and samples were sent for smear and culture. Blood culture at the time of presentation was negative, but urine analyses showed abundant red blood cells and white blood cells, and ovoid yeast forms were observed. Based on the basis of these findings, urinary tract infection and fungemia associated with lithotripsy was suspected. Vitrectomy and voriconazole injection of the left eye was performed. Systemic voriconazole was given intravenously. The vitreous cultures confirmed our suspicions and were positive for C. albicans. According to the result of antimicrobial susceptibility test, intravitreal injection of amphotericin-B (0.5 µg/0.1 mL) was performed. Postoperatively, the patient was treated with 200 mg fluconazole intravenously daily, for 2 weeks. The patient’s best corrected visual acuity in the affected eye was 20/40 in follow up.

3 years later, the patient presented to the hospital again with blurred vision and floaters in his right eye for 3 days. His best corrected visual acuity was 20/400 (right) and 20/40 (left). The clinical presentation of his right eye was identical to the left eye 3 years ago. Like 3 years ago, the patient also received ureteroscopy and HLL in the same hospital 2 weeks before. The treatment was similar and his best corrected visual acuity in the right eye was also 20/40 in follow up.

### Case 2

A 31-year-old female, in her 30th week of pregnancy, presented with a 10-day history of sudden and worsening vision loss with dark sports in both eyes. Her best corrected visual acuity was 20/1000 in both eyes. She had a history of high fever of 40.5 ℃ 2 weeks ago for unclear reason, and receiving three doses of intravenous injections of antibiotics prior to her visual complaints. On examination, she was not febrile and without obvious systemic symptoms, but she had no perception of light in the affected eye. There was circumcorneal congestion and 3 + cells in the anterior chamber. Fundus examination revealed 3 + anterior vitreous cells, cotton ball-like opacities in the vitreous with subretinal exudates and retinal hemorrhages. The patient’s G test for β-d-glucan was 218.9pg/ml. The patient’s blood culture and vaginal discharge smear showed positive for C. albicans, but her vitreous tap was negative for culture. Based on her clinical presentation, candida endophthalmitis was suspected. 100ug of Voriconazole was given intravitreal twice on Day 1 and Day 4, with 50 mg of Amphotericin B was used intravenously for 3 days. However, her visual acuity continued to drop and the patient underwent vitrectomy and 30 mg of amphotericin-B was injected intravenously for 3 days. The patient recovered gradually, and 200 mg fluconazole was taken orally per day for 10 days. Her VA was 20/60 on day 14 in the follow up, and the patient had urgent c-section on day 16 because of sudden lower abdominal pain and fetal distress. 1% prednisolone acetate and 0.2% fluconazole eye drops were used four times per day in the follow up, and her VA recovered to Vod 20/60, Vos 20/25 on day 40.

### Case 3

A 46-year-old man was transferred from intensive care unit (ICU) to the ophthalmology department with a 1-week history of painful vision loss in the right eye. His VA was light perception (LP, right) and 20/25 (left). Examination of the right eye showed diffused conjunctival injection, cluster of keratic precipitates (KP), anterior chamber inflammatory cells, rubeosis iridis, posterior synechia and a dense cataract. The intraocular pressure was 34mmHg (right) and 10mmHg (left). The patient had invasive liver abscess syndrome (ILAS) and underwent percutaneous transhepatic drainage of the liver segment 2 weeks ago. The patient’s blood, urine and sputum cultures showed K. pneumoniae infection, and the hypervirulent K1 serotype was identified on a polymerase-chain-reaction assay. He had tracheal intubation due to respiratory failure, and he was hospitalized in the ICU for 2 weeks because of poor systemic conditions with ventilator requirements. He was found to have type 2 diabetes mellitus during this period. EE with retinal detachment (RD) of the right eye was diagnosed, and emergency vitrectomy was performed. Vitreous sample was sent for culture and 2 mg of Ceftazidime was injected intravitreally. The culture of the vitreous sample also showed K. pneumoniae. The patient received intravenous injection of 2 g Ceftazidime for another 7 days and his VA was LP in the follow up.

## Discussion and conclusions

EE is usually caused by spreading of systemic infections, and the causative pathogens are mostly fungi or bacteria [7]. Debilitated patients, especially those who are immunodeficient are more susceptible to this disease. In patients with severe systemic of infections, their ocular symptoms may be underestimated or even unnoticed unless specifically asked for or checked by physicians [6]. When patients with systemic infection develop painful red eyes or vision deterioration, non-ophthalmologists should have the knowledge of the potential of EE, and seek advice from ophthalmologists promptly [6]. 

In case 1, the patient has endogenous candida endophthalmitis (ECE), which is a rare complication of urologic procedures, such as holmium laser lithotripsy and extracorporeal shock wave lithotripsy (ESWL). C. albicans is the most common cause of EE [9]. Under normal conditions, C. albicans should not colonize inside the urinary tract. However, C. albicans could present in urine ( i.e. Candiduria), which may occur in both asymptomatic and symptomatic urinary tract infections [10]. During the urologic procedures, even though minimal invasive, C. albicans in the urinary tract may enter bloodstream due to mechanical abrasion and epithelial trauma, seed into the eyes and lead to intraocular candidiasis [11]. 

Most of these cases happened in patients with predisposing factors, such as diabetes mellitus, long-term systemic antibiotic usage, hospitalization, etc. In our case, the patient had two onsets of unilateral ECE, and both happened 2 weeks after being hospitalized for HLL. C. albicans was observed in his urine sample without any symptoms. Because asymptomatic candiduria is mostly benign and is not counted as a definite disease, the patient did not receive any prophylactic antibiotics or antifungal before and after HLL. However, based on his past history of ECE after HLL, it may be important to apply prophylactics therapy in patients who are susceptible to or who have past history of EE.

Case 2 is also ECE, but it is an extremely rare condition of invasive candidiasis induced EE during pregnancy. Invasive candidiasis is associated with high morbidity and mortality [12], and both the diagnosis and treatment were a challenge for physicians in this case. This patient was a pregnant woman and the treatment requires multidisciplinary management. For non-ophthalmologist physicians, early diagnosis of the disease is critical for the prognosis. In this case, the diagnosis is confirmed by positive blood culture and G-test for β-D-glucan, which has good specificity for the diagnosis of invasive candidiasis [13, 14]. However, her vitreous tap culture was negative for either fungus or bacteria. To be noted here that the negative result of vitreous tap culture should be interpreted with caution, because fungi are difficult to grow in culture. In addition, C. albicans primarily locates within inflammatory nodules, thus may escape from one-time sampling and thus cause negative result. According to a study evaluating the 31 case series published between 2011 and 2020, the positive vitreous culture rates varies between 70.7% and 30% [15]. If polymerase chain reaction (PCR) test is available, it is more accurate and time efficient for etiological diagnosis [9]. 

It was unclear where the candidemia originated since the patient had no past medical history. We noted that the patient’s vaginal discharge smear showed positive for C. albicans, which is the most common agent what causes lower genital tract infection during pregnancy [16]. In rare cases, ascending vaginal C. albicans infection from the lower maternal genital tract may occur, causing adverse pregnancy outcomes such as preterm birth, abortion and premature rupture of membranes, which may provide a transmission route for pathogens in to the blood stream [16, 17]. 

Case 3 is EE cause by ILAS, which is a newly reported syndrome characterized by liver abscess and metastatic infection caused by K. pneumoniae [18]. EE is a relative common extrahepatic complication of ILAS and the diagnosis should be considered in such patients with acute vision loss. Some patients may even first present with visual changes without abdominal complaints.

In this case, the patient was too debilitated in the ICU and his treatment strategy was focused on life saving during the acute phase of ILAS. This patient had RD, which is a complication of endophthalmitis that is also vision threatening. It is reported that the incidence of RD after endophthalmitis was 14.8% [19]. The therapeutic paradox is that the patient who undergoes RD surgery always need to remain a face-down position, but this is a challenge for weak patients with systemic conditions. When physicians come across such treatment dilemma, it is important for ophthalmologists and non-ophthalmology doctors to work together to evaluate the severity of both the systemic and eye conditions, and make individualized treatment strategy.

We presented 3 typical EE cases from 2 medical centers with different special systemic conditions, and their onset of ophthalmic symptoms were secondary to their primary medical issues. There are a lot of patients with EE who have ocular symptoms as the initial presentation and are found to have hidden infection at other sites later. These patients are usually treated appropriated by ophthalmologists in time. If the patients have a systemic presentation first, and are specifically educated for the possibility of EE and asked for the eye symptoms, they may be referred to the ophthalmology department at earlier onset. From the perspective of physicians, the choice of appropriate initial treatment could also help to lower the possibility of EE. Because of the blood–retinal barrier, the penetration of antiinfection drugs into the posterior segment of the eye after systemic administration is limited [20]. If a patient has higher chance of developing EE, such as the patient with ILAS in case 3, physicians need to pay more attention to drug selections to control both systemic and eye infections. Ignoring eye involvement during the treatment of infections at other sites can lead to insufficient treatment of latent ocular infection. EE may present sometime after initial systemic illness and lead to severe impairment to patients’ visual health and quality of life. As in case 1, we need to learn the lesson and do not stumble over the same “stone” again.

### Electronic supplementary material

Below is the link to the electronic supplementary material.

Supplementary Material 1

Supplementary Material 2

## Data Availability

No datasets were generated or analysed during the current study.

## Abbreviations

ECE: endogenous candida endophthalmitis
EE: endogenous endophthalmitis
ESWL: extracorporeal shock wave lithotripsy
HLL: holmium: YAG laser lithotripsy
ICU: intensive care unit
ILAS: liver abscess syndrome
KP: keratic precipitates
NEDS: Nationwide Emergency Department
OCT: optical coherence tomography
PCR: polymerase chain reaction
RD: retinal detachment
