# Supplementary material for: Do not stumble over the same “stone” twice: a case series of endogenous endophthalmitis secondary to severe systemic diseases
Source: BMC Ophthalmol. 2024 May 17;24:214. doi: 10.1186/s12886-024-03478-7 (PMC11100056; doi:10.1186/s12886-024-03478-7)
Supplement: Supplementary file 2 — Supplementary Material 2 [file 12886_2024_3478_MOESM2_ESM.pdf]

BioMed Central

Consent form

I Changjun Lu;Jun Wang;Cui Liu [Name] give my consent for information about myself/my child or ward/my relative (circle as appropriate) to be published in BMC Ophthalmology, Do not stumble over the same “stone” twice: a case series of Endogenous Endophthalmitis secondary to severe systemic diseases;Ying He; Weijuan Zeng; Wenjian Shi

[Name of journal, manuscript number and corresponding author].

I understand that the information will be published without my/my child or ward’s/my relative’s (circle as appropriate) name attached, but that full anonymity cannot be guaranteed.

I understand that the text and any pictures or videos published in the article will be freely available on the internet and may be seen by the general public. The pictures, videos and text may also appear on other websites or in print, may be translated into other languages or used for commercial purposes.

I have been offered the opportunity to read the manuscript.

Signing this consent form does not remove my rights to privacy.

Name: Changjun Lu;Jun Wang;Cui Liu

Date: 2024/3/10

Signed: Changjun Lu Jun Wang Cui Liu

Author name: Min Ke;Yingying Gao

Date: 2024/3/10

Signed: Min Ke Ying Ying Gao

Please keep this consent form in the patient’s case files. The manuscript reporting this patient’s details should state that ‘Written informed consent for publication of their clinical details and/or clinical images was obtained from the patient/parent/guardian/ relative of the patient. A copy of the consent form is available for review by the Editor of this journal.
